# Public Health Education and Training in Viet Nam: Adaptations Are Needed for Better Responses to New Public Health Needs

**DOI:** 10.3389/phrs.2025.1608066

**Published:** 2025-02-10

**Authors:** Nguyen Thanh Ha, Bui Thi Tu Quyen, Le Thi Vui, Do Thi Hanh Trang, Nguyen Thai Quynh Chi, Le Quang Cuong, Hoang Van Minh

**Affiliations:** ^1^ Department of Nutrition and Food Safety, Faculty of Environmental and Occupational Health, Hanoi University of Public Health, Hanoi, Vietnam; ^2^ Department of Biostatistics, Faculty of Fundamental Science, Hanoi University of Public Health, Hanoi, Vietnam; ^3^ Department of Population- Reproductive Health, Faculty of Social and Behavioral Sciences, Hanoi University of Public Health, Hanoi, Vietnam; ^4^ Department of Academic Affairs, Faculty of Environmental and Occupational Health, Hanoi University of Public Health, Hanoi, Vietnam; ^5^ Department of Health Promotion, Faculty of Social and Behavioral Sciences, Hanoi University of Public Health, Hanoi, Vietnam; ^6^ Department of Neurology, Hanoi Medical University, Hanoi, Vietnam; ^7^ Department of Health Policy and Economics, Health Management Training Institute, Hanoi University of Public Health, Hanoi, Vietnam

**Keywords:** Essential Public Health Functions, education, public health, adaptation, Vietnam

Public health has been defined as “*the science and art of preventing disease, prolonging life, and promoting health through the organized efforts of society*” [1]. Public health plays a crucial role in improving community health by achieving goals related to disease prevention, promoting the implementation of beneficial behaviors to reduce the risk of infectious, non-communicable, and injury-related diseases, and ensuring that the community has access to healthcare services of good quality [2–5].

According to the World Health Organization (WHO), public health has 12 Essential Public Health Functions (EPHFs), the minimum requirements for Member States to ensure community health in a comprehensive, integrated, and sustainable manner [3, 4]. In 2024, WHO recommended competencies in 20 core areas across six domains, with 74 specific and 28 leadership behaviors [6]. In the latest update, the WHO has added three new functions: EPHF2 (*Public health emergency management); EPHF5 (Health protection), and EPHF12 (Access to and utilization of health products, supplies, equipment, and technologies)* [6]. At the same time, it has emphasized equity in the quality function and in the provision of health services and Multisectoral planning in the function of Multisectoral planning financing and management for public health [6]. This framework has guided many countries in developing competency-based public health training programs.

In Vietnam, public health education began in 2001 with the establishment of the Hanoi School of Public Health (HSPH), and became one of the first universities to offer the Bachelor of Public Health (BPH) in Vietnam. National competency standards for BPH were developed based on international references, local regulations, and Vietnam’s public health context [7, 8]. These include eight standards, focusing on public health science, analysis, policy development, communication, leadership, and cultural diversity. Each standard has specific criteria for evaluation, totaling 50 criteria, with 30 core ones tailored to Vietnam’s public health needs [9].

Nowadays, public health education and training in Vietnam have been more widely implemented nationwide. BPH, Master of Public Health (MPH), and PhD in Public Health (PhDPH) training programs have been developed and conducted by the Hanoi University of Public Health (formerly the HSPH, which is the unique training institution specialized in public health in Vietnam) and faculties or departments of Public Health within nearly 20 medical universities/schools in the country.

In general, compared to the WHO’s EPHFs 2024 [6], all of the three formal training programs (BPH, MPH, and PhDPH) have fully covered the subfunctions of EPHFs 1, 6, 7, and 8 [6] (i.e., *Public health surveillance and monitoring, Disease prevention and early detection; Health promotion; and Community engagement and social participation*).

The BPH training program meets 10 of 12 EPHFs and 27 of 48 subfunctions in the WHO’s 2024 EPHF [6] list. It does not fully address EPHF9 (*Public health workforce development*) and EPHF12 [6]. Minimal coverage is noted in EPHF2 (1/5 subfunctions), EPHF3 (1/4 subfunctions), and EPHF4 (2/5 subfunctions) [6], indicating gaps in emergency preparedness, governance, and cross-sectoral planning.

The MPH training program addresses 11 of 12 EPHFs and 32 of 48 subfunctions [6], falling short in EPHF12 [6]. Compared to the BPH program, it covers five additional subfunctions. These include subfunction 4.2 in EPHF4 [6] (promoting cross-sectoral public health planning with WHO’s Health in All Policies approach) and subfunction 5.1 in EPHF5 [6] (developing and enforcing regulatory frameworks for protecting populations from health hazards). In EPHF9 [6], the MPH program fulfills subfunction 9.1 [6] (planning and evaluating the public health workforce) and subfunction 9.2 [6] (developing workforce education and training across competencies, including intersectoral work and emergency response). Additionally, it addresses subfunction 10.2 in EPHF10 [6] (promoting equity in access to health and social care services). These achievements highlight the MPH program’s focus on cross-sectoral collaboration, workforce development, and equity promotion. However, addressing EPHF12 [6] remains a critical area for improvement.

The PhDPH training program addresses 11 of 12 EPHFs [6] and 36 of 48 subfunctions but does not meet EPHF12 [6]. Compared to the MPH program, the PhDPH program covers four additional subfunctions. These include subfunction 2.2 in EPHF2 [6] (planning and building capacity for public health emergency preparedness and response with intersectoral collaboration) and subfunction 2.5 [6] (engaging communities and stakeholders across sectors for public health emergency management). In EPHF3 [6], the PhDPH program also addresses subfunction 3.3 [6] (developing, monitoring, and evaluating public health regulations and laws as frameworks for governance and services). Additionally, it fulfills subfunction 11.3 in EPHF11 [6] (promoting integrating public health operational research into broader research agendas). The PhDPH program demonstrates greater alignment with emergency preparedness, governance, and research prioritization than the MPH program. However, addressing EPHF12 [6] remains a shared gap across all training programs.

Of the three new EPHFs added in 2024 (EPHFs 2, 5, and 12 [6]), none of the training programs address EPHF12 [6]. The MPH and PhDPH programs meet all three subfunctions of EPHF5 [6], while the BPH program does not fulfill subfunction 5.1 [6] (developing and evaluating regulatory frameworks to protect populations from health hazards). Regarding EPHF2 [6], the BPH and MPH programs meet only subfunction 2.1 (monitoring and analyzing public health information to identify and prioritize risks, including emergencies). In contrast, the PhDPH program addresses three of the five subfunctions of EPHF2 [6]. These include subfunction 2.1, subfunction 2.2 [6] (planning and building capacity for emergency preparedness and response as part of routine health systems with intersectoral collaboration, including a national emergency response operations plan), and subfunction 2.5 [6] (engaging communities and stakeholders across public, private, and allied sectors in a whole-of-society approach to emergency management). While progress is evident in EPHFs 2 and 5, the gap in addressing EPHF12 [6] highlights an area for improvement in all training programs, particularly in ensuring access to and utilization of essential health products, supplies, and technologies. The findings underscore the need for further curriculum alignment with the comprehensive EPHF framework.

The above comparisons between the public health education and training programs in Vietnam with the requirements of WHO’s EPHFs in 2024 [6] highlight the need for further adaptation of these programs in Vietnam. These comparisons also serve as a basis to help universities propose improvements to the existing training programs and develop future short-term courses to respond to the changing public health context after the COVID-19 pandemic, globalization, and the digital era.

At the macro level, an overall review of the WHO’s EPHFs 2024 [6] is needed to identify the functions and services that the public health workforce in Vietnam can perform appropriately in the given context. According to the WHO [10], it is recommended to identify the current status of EPHF implementation, consider EPHFs in the health sector and other related sectors, and identify workforce groups that can perform part or all of the EPHFs to achieve universal health coverage, health security, and health improvement. This is also an opportunity for Vietnam to review the competency standards of BPH to adjust the necessary competencies for those workforce groups so that they can respond to the changes following the COVID-19 pandemic.

At the micro level, education and training institutions in Vietnam must review the program learning outcomes and the curriculum contents of the three existing degree programs to adjust and align them with EPHFs 2024 [6]. Priority should be given to adjusting the regular public health degree programs for the core public health workforce. Furthermore, universities can develop short-term courses for other relevant workforce groups that can provide public health services based on their functions and responsibilities.
